# Comparative genome sequencing identifies a prophage-associated genomic island linked to host adaptation of *Lawsonia intracellularis* infections

**DOI:** 10.1186/1297-9716-44-49

**Published:** 2013-07-04

**Authors:** Fabio A Vannucci, Molly R Kelley, Connie J Gebhart

**Affiliations:** 1 Department of Veterinary and Biomedical Science, College of Veterinary Medicine, University of Minnesota, St. Paul, MN, USA

## Abstract

*Lawsonia intracellularis* is an obligate intracellular bacterium and the causative agent of proliferative enteropathy (PE). The disease is endemic in pigs, emerging in horses and has also been reported in a variety of other animal species, including nonhuman primates. Comparing the whole genome sequences of a homologous porcine *L*. *intracellularis* isolate cultivated for 10 and 60 passages in vitro, we identified a 18-kb prophage-associated genomic island in the passage 10 (pathogenic variant) that was lost in the passage 60 (non-pathogenic variant). This chromosomal island comprises 15 genes downstream from the prophage DLP12 integrase gene. The prevalence of this genetic element was evaluated in 12 other *L*. *intracellularis* isolates and in 53 infected animals and was found to be conserved in all porcine isolates cultivated for up to 20 passages and was lost in isolates cultivated for more than 40 passages. Furthermore, the prophage region was also present in 26 fecal samples derived from pigs clinically affected with both acute and chronic forms of the disease. Nevertheless, equine *L*. *intracellularis* isolates evaluated did not harbor this genomic island regardless of the passage in vitro. Additionally, fecal samples from 21 clinically affected horses and four wild rabbits trapped in horse farms experiencing PE outbreaks did not show this prophage-associated island. Although the presence of this prophage-associated island was not essential for a virulent *L*. *intracellularis* phenotype, this genetic element was porcine isolate-specific and potentially contributed to the ecological specialization of this organism for the swine host.

## Introduction

*Lawsonia intracellularis* is a gram-negative obligate intracellular bacterium and the etiologic agent of proliferative enteropathy (PE). The disease is characterized by thickening of the intestinal epithelium due to enterocyte proliferation which is directly associated with the presence of intracytoplasmic bacteria [1]. PE is endemic in pigs, an emerging disease in horses and is also described in various other species, including nonhuman primates, wild mammalians and ratite birds [2-4].

PE was first reported in 1931 [5], but the causative bacterium was isolated in 1991 from hamsters and in 1993 from pigs using cell culture under strict microaerophilic environmental conditions [6,7]. To date, growth of the bacteria in axenic (cell-free) media has not been reported. The fastidious properties related to the isolation and cultivation of *L*. *intracellularis* has limited molecular studies comparing bacterial isolates, so far, there are approximately 15 isolates worldwide. The whole genome of a porcine *L*. *intracellularis* isolate (PHE/MN1-00) has been sequenced and annotated (accession: PRJNA183) using Sanger-based sequencing and a total of 1 719 014 base pairs distributed into one small chromosome (1.46 Mb) and three plasmids. The presence of few potential virulence factor-encoding genes identifying by comparative sequence analysis and many hypothetical proteins suggest that this organism has adopted mechanisms of survival and pathogenesis that are unique among bacterial pathogens [1].

Non-pathogenic isolates obtained through multiple passages in cell culture have not been successful at inducing typical PE lesions or reversing virulence in experimentally-infected pigs [8]. Conversely, clinical and subclinical disease has been previously reproduced using cultivated *L*. *intracellularis* at low passages (4 to 20) in cell culture [9-12]. Vannucci et al. recently described attenuation of the virulence properties between 20 and 40 cell passages in vitro, but the molecular mechanisms responsible for this phenotype remain unclear [13]. The loss of pathophysiological properties of *L*. *intracellularis* associated with its adaptation to in vitro conditions due to mutational events at the DNA level has been speculated [13]. However, standard DNA-based typing techniques, such as pulsed field gel electrophoresis (PFGE), multilocus sequence typing (MLST) and variable number tandem repeat (VNTR) have shown identical genotypes in both pathogenic (low passage) and non-pathogenic (high passage) variants [14-16]. A more comprehensive genomic analysis of these two phenotypic variants is crucial to determine genomic variations which might potentially be associated with virulence properties. In addition, the characterization of genomic variations may reveal DNA sequence markers useful for studying genetic variations among clinical samples of *L*. *intracellularis* without requiring bacterial isolation.

The present study used high-throughput DNA sequencing technology to compare and characterize genomic variations between homologous pathogenic (passage 10) and non-pathogenic (passage 60) *L*. *intracellularis* variants. The prevalence of a prophage-associated genomic island which was present in the pathogenic but not in the non-pathogenic variant was evaluated in porcine and equine isolates, as well as in fecal samples positive for the presence of *L*. *intracellularis* DNA derived from infected animals.

## Materials and methods

### *L*. *intracellularis* isolate and growth conditions

The porcine *L*. *intracellularis* isolate PHE/MN1-00 previously obtained from a pig with the hemorrhagic form of PE was used [10]. A previous study using experimentally-infected pigs confirmed the pathogenic and non-pathogenic properties of these two variants [13]. Murine fibroblast-like McCoy cells were grown in Dulbecco’s Modified Eagles Medium with 1% L-glutamine, 7% fetal bovine serum and 0.5% amphotericin B without antibiotics [17]. Pure culture of the bacteria at passage 6 (pathogenic variant) and 56 (non-pathogenic variant) was thawed and grown in McCoy cells (ATCC CRL 1696) for three continuous passages in order to allow the bacteria to recover from the frozen storage. T_75_ cell culture flasks containing one-day-old McCoy cells (30% confluence) were infected with bacterial suspensions containing approximately 10^4^ *L*. *intracellularis* organisms.

After a recovery period, pure cultures of *L*. *intracellularis* at passages 10 and 60 were prepared and used for DNA sequencing. Inoculated cultures were placed in an anaerobic chamber which was evacuated to 500 mmHg and refilled with hydrogen gas. Infected cultures were then incubated for seven days in a Tri-gas incubator with 83.2% nitrogen gas, 8.8% carbon dioxide, 8% oxygen gas and a temperature of 37°C, as previously described [6]. During each passage, the cell culture infection was monitored by counting the number of heavily infected cells (HIC) using immunoperoxidase staining with polyclonal antibody specific for *L*. *intracellularis*[18]. All procedures used in the present study were approved by the Institutional Sponsored Projects Administration of the University of Minnesota.

### DNA preparation and genome sequencing

Bacterial cultures were prepared for extraction and sequencing using the supernatants of infected cell cultures in order to minimize the presence of eukaryotic DNA and enhance the purity of the bacterial suspension for DNA extraction. Briefly, cell culture supernatants were passed t through 0.8 μm sterile filters to remove potential detached McCoy cells present in the cell monolayer supernatant. Filtered bacterial suspensions were then pelleted by centrifugation at 8000 for 20 min. The pellets were used for DNA extraction using DNeasy Blood & Tissue Kit® (Qiagen, Valencia, CA, USA), according to the manufacturer’s instructions.

The sequencing procedures were conducted through the core facility of the Biomedical Genomics Center at the University of Minnesota. Following quantification using the PicoGreen Assay of the DNA generated in the library preparation, the samples were loaded on the Illumina® Genome Analyzer GA IIx platform with paired reads of 76 bp. Base calling and quality filtering were performed following the manufacturer’s instructions (Illumina® GA Pipeline, San Diego, CA, USA).

### Genome assembly and comparison

The sequence contigs were generated using Velvet assembly software [19] followed by alignment onto the reference genome using the Sequencer® 5.0 (Gene Codes Corporation, Ann Arbor, MI, USA). The assembly visualization was performed using Table software which allowed comparative genome analysis, evaluation of the genome coverage and the identification of single nucleotide substitutions (SNPs), insertions and deletions (indels) [20]. Based on the sequence visualization, manual annotation, manipulation and comparison were performed between both variants. Indels and SNPs previously identified in the comparative visualization analysis were confirmed by Sanger-based sequencing of directed PCR amplicons generated from the corresponding regions.

### Prevalence of the prophage-associated genomic island *L*. *intracellularis* isolates

The presence of the prophage-associated genomic island identified by whole-genome sequencing in the pathogenic variant (passage 10) of the porcine *L*. *intracellularis* isolate PHE/MN1-00 but not in the non-pathogenic variant (passage 60) was evaluated in 12 other isolates from our collection at low and high passages (Table 1). Additionally, a total of 53 fecal samples submitted to the Minnesota Veterinary Diagnostic Laboratory from different states of the United States, which had previously tested positive for the presence of *L*. *intracellularis* DNA by PCR, were evaluated. The positivity of these samples was confirmed concurrently with the PCR analysis to determine the presence of the prophage genes. A total of 26 fecal samples were collected from naturally infected pigs in seven states (Iowa, Illinois, Minnesota, Missouri, Nebraska, Oklahoma and North Carolina), along with samples from 21 naturally infected horses from eight states (Florida, California, Iowa, Kentucky, Minnesota, Tennessee, Texas and Virginia) and four wild rabbits captured in a California horse breeding farm experiencing clinical cases of *L*. *intracellularis* infections.

**Table 1:** **Summary of the *L. intracellularis *isolates used in the study to evaluate the presence of the prophage-associated genomic island**

**Strain ID**	**Origin of the strains**		**Number of passage**	**Presence of prophage**
	**Host species**	Country*		
PHE/MN1-00	Swine	USA	10	+
			20	+
			40	-
NWumn05	Swine	USA	20	+
			40	-
VPB4	Swine	USA	10	+
			>150	-
GBI06	Swine	USA	10	+
DBumn06	Swine	USA	10	+
D15540	Swine	DK	20	+
PHE-BR	Swine	BR	10	+
963/3	Swine	UK	80	-
916/91	Swine	UK	60	-
LR189/5/83	Swine	UK	90	-
Foal96	Equine	USA	> 100	-
E40504	Equine	USA	10	-
Ham1	Hamster	USA	> 100	-

* *USA*, United States of America, *DK*, Denmark, *BR*, Brazil, *UK*, United Kingdom.

Specific primers targeting all 15 genes included in the prophage-associated genomic island and the two flanking genes present immediately before and after the prophage region were designed (Table 2). DNA from fecal samples was extracted using QIAamp® DNA Stool Mini kit (Qiagen) and the protocol was performed according to the manufacturer’s instructions. PCR reactions were performed in a 25 μL volume using HotStar®Taq DNA polymerase (Qiagen) and 0.6 μM of each primer under the following conditions: 95°C for 15 min, 35 cycles (94°C/30 s, 52°C/60 s, 72°C/30 s) and final extension at 72°C for 10 min.

**Table 2:** **Primers targeting the prophage-associated genomic island of porcine *L. intracellularis *isolates and the two flanking genes**

**Gene**	**Gene product**	**Entrez gene ID**	**Primers (5′ → 3′)**
LI0172*	Hypothetical protein	4059866	ATTGATGCTCCTGTCCCACG
			ACCACATGGTGGATTCGTCC
LI0173	Prophage DLP12 integrase	4059867	CGTCGTATTCTGCGCTTTGG
			ATCATCAGCTACACGAGCGG
LI0174	Hypothetical protein	4059868	CAGGAAGATGCTGTGTGGCT
			ATTCGCTTTCGCAATACGGC
LI0175	Hypothetical protein	4059869	CCCACGGACGAAGACTTTGA
			TCAGCTTTCGGGCATGGATT
LI0176	Hypothetical protein	4059870	ACAGACCTCTATGCTCCCGT
			TCAGCGTCTTGGGGCTTTAG
LI0177	Hypothetical protein	4059871	ACACCACCATTACCACTGCT
			ACACCACCATTACCACTGCT
LI0178	Hypothetical protein	4059872	TTCCTCCTGCGTGTCGTAAC
			ATTTCTCCCTGGCTCTGCAC
LI0179	Ribosomal protection tetracycline resistance protein	4059813	CGTCAGCAAAGCGGAAACAA
			AAACGGCCTTGGCATTCAAC
LI0180	Hypothetical protein	4059873	GAACCGGTGAGCCAAGTGTA
			TTCCTTCGGGAGTCGAGGAT
LI0181	Endonuclease I	4059874	AGGCTAAGCGCATACTGCAA
			CAGCATTGACAGACCCGACT
LI0182	Recombination protein-phage associated	4059875	TGGATTTCCAGCACAGCCAT
			TGAACCGTCCTGAAGCTCAC
LI0183	Hypothetical protein	4060170	CTCTCGACGCATCTTCCCTC
			TCCATTCCGCCGTCATGAAA
LI0184	Hypothetical protein	4060171	TTGGACTGGCTCTTACGCAG
			GATGAAGCCCACGTCAGGAA
LI0185	MoxR-like ATPases	4060172	GTCATGCGTCAGAACATCGC
			CCTGTTTGGAGAGAGGCTGG
LI0186	Hypothetical protein	4060173	CCTTCCTGGGCCAACATCAT
			CACGCTTGGGCATATTTCCG
LI0187	Hypothetical protein	4060174	ATCGGTTCTTCGGATACCGC
			GAATCCTGCGTAGATCGGCA
LI0188*	ATP-dependent Zn proteases (*ftsH*)	4059755	GAGCTGTAGCTGGTGAAGCA
			ACGTGCAACAAGTGCATGTC

* Flanking genes located before and after the prophage-associated genomic island.

## Results

### Genome comparison between porcine *L*. *intracellularis* isolate (PHE/MN1-00) at low and high passages

The present study resequenced the isolate PHE/MN1-00 which was used to generate the reference genome of *L*. *intracellularis* available at the National Center for Biotechnology Information (NCBI accession: PRJNA61575) using Sanger-based sequencing. Although there is no information available regarding the number of passages in vitro used in this first sequencing project, our results showed identical DNA contents using the passage 10. This strongly indicates that the first genome project used low passage-cultivated bacteria.

The whole genome sequencing generated 83 999 466 and 128 258 573 millions of bp for *L*. *intracellularis* cultivated at 10 and 60 passages in vitro, respectively. For the passage 10, the average coverage of 42X was assembled with mean contig length of 11 845 bp (minimum: 2175 and maximum: 112 744) and 14.1% of unused data which represented eukaryotic-derived DNA. The data from the passage 60 (NCBI accession: PRJNA61575) generated an average coverage of 66X assembled with mean contig length of 10 674 bp (minimum: 1189 and maximum: 119 534) and 11.3% of eukaryotic -derived DNA.

The comparative analysis between homologous pathogenic (passage 10) and non-pathogenic (passage 60) *L*. *intracellularis* variants reveals a major deletion of 18,086 base pairs (bp) in the passage 60 which includes 15 genes starting from the prophage DLP12 integrase gene (LI0173). General features of the genome comparison are summarized in Table 3. In addition to the prophage region, only four single nucleotide polymorphisms (SNPs) were identified in the chromosome and one in the plasmid C of the passage 60 variant (Table 4). DNA sequences from plasmids A and B were identical in both the passage 10 and 60 variants.

**Table 3:** **General characteristics of the two *Lawsonia intracellularis *homologous variants**

***L. intracellularis *PHE/MN1-00**	**Passage 10**	**Passage 60**
Chromosome size	1 457 619	1 439 533
G° + °C content (%)	33.3	33.1
Total number of protein-coding genes	1183	1168
Prophage element	1 (18.1 Kb)	0
Plasmids	3	3
Variant-specific genes	15	0

**Table 4:** **Single nucleotide substitutions in passage 10 and passage 60 of the porcine *L. intracellularis *isolate PHE/MN1-00**

**Gene locus (Position)**	**Description**	**Amino acid change**		**Substitution**
		**Passage 10**	**Passage 60**	
*Chromosome*				
LI0064 (86 203)	Transcriptional regulator	Glutamic acid	Lysine	Non-synonymous
		(**G**AA)	(**A**AA)	
LI0473 (577°207)	DNA methylase	Lysine	Glutamic acid	Non-synonymous
		(**A**AA)	(**G**AA)	
LI0532-LI0533 (651°651)	Intergenic region			
LI0905 (1°129°621)	DNA-directed RNA polymerase	Lysine	Glutamic acid	Non-synonymous
		(**A**AG)	(**G**AG)	
*Plasmid C*				
LIC103 (190°854)	Methyl-accepting chemotaxis	Cysteine	Cysteine	Synonymous
		(TG**C**)	(TG**T**)	

Bold underline letters: Single nucleotide substitution from passage 10 to passage 60.

### Characterization of the *L*. *intracellularis* prophage-associated genomic island

**Figure 1:**
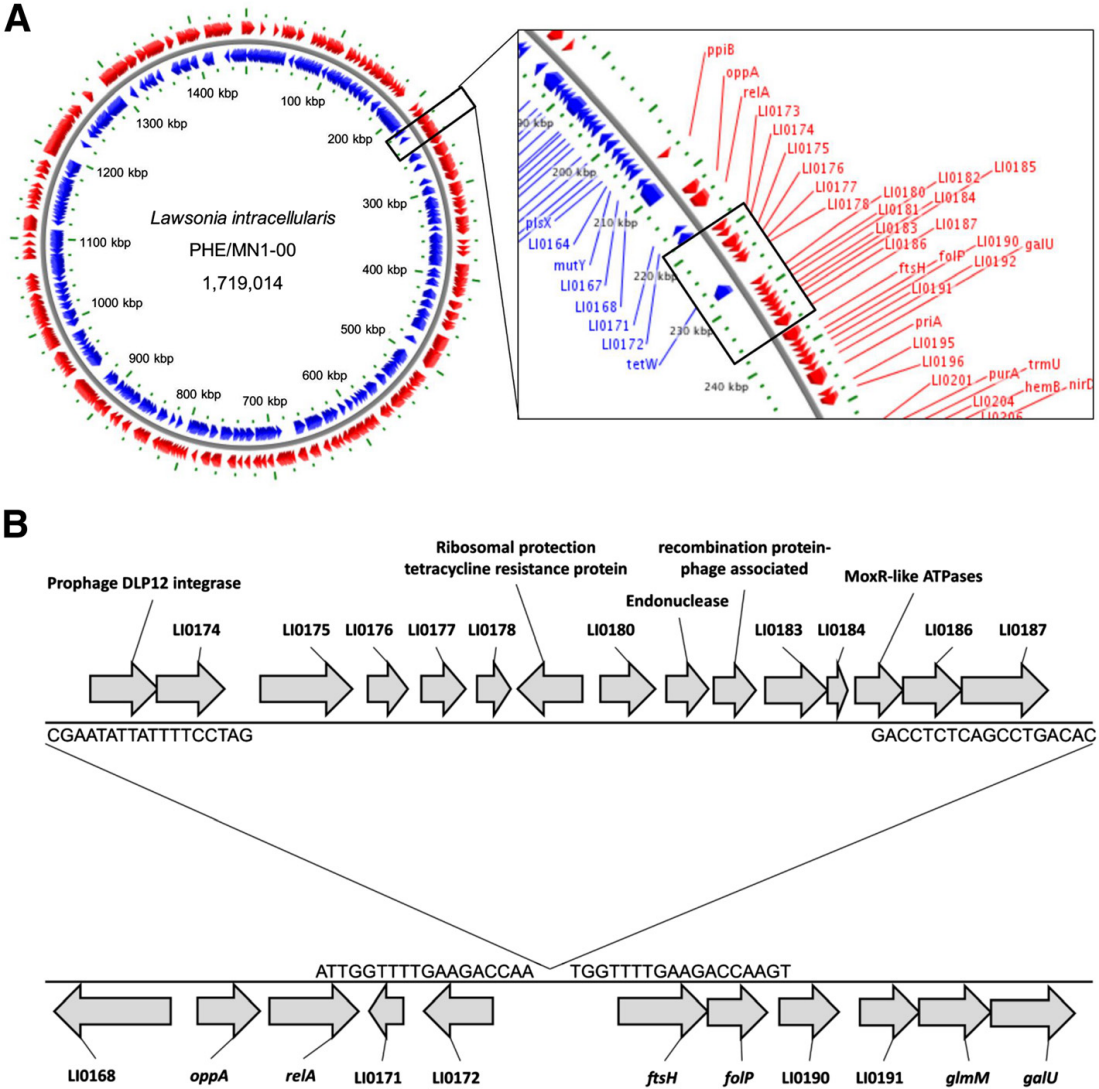
**Prophage DLP12-associated genomic island. (A)** Chromosome map of the porcine *L*. *intracellularis* isolate PHE/MN1-00 at passage 10 showing the genomic location of the prophage element. **(B)** Genetic organization of the prophage region (from prophage DLP12 integrase to LI0187 gene). Loci between LI0172 and *ftsH* gene from where the prophage excised and is no longer present in the equine and rabbit-derived isolates and in the porcine *L*. *intracellularis* isolate PHE/MN1-00 at passage 60.

A prophage-associated genomic island located in the chromosome represented the major genomic deletion identified in the porcine *L*. *intracellularis* isolate PHE/MN1-00 at passage 60. This deletion is located at the position 218 578 to 236 664 and comprises 15 genes starting from the prophage DLP12 integrase gene (Figure 1). Besides this prophage integrase, four more genes with known function have been annotated within the region: ribosomal protection tetracycline resistance protein (LI0179); endonuclease (LI0181); recombination protein-phage associated (LI0182) and MoxR-like ATPases (LI0185). The other ten genes were predicted to encode hypothetical proteins. Sequence analyses of these genes using the Kyoto Encyclopedia of Genes and Genomes (KEGG) database reveals the presence of six motif sequences: three proteins (LI0177, LI0184 and LI0186) with unknown functions but previously reported in other bacterial organisms; ferric reductase NAD binding domain (LI0174); RNA polymerase III subunit RPC82 helix-turn-helix domain (LI0175) and cobalamin biosynthesis protein CobT (LI0187).

**Figure 2:**
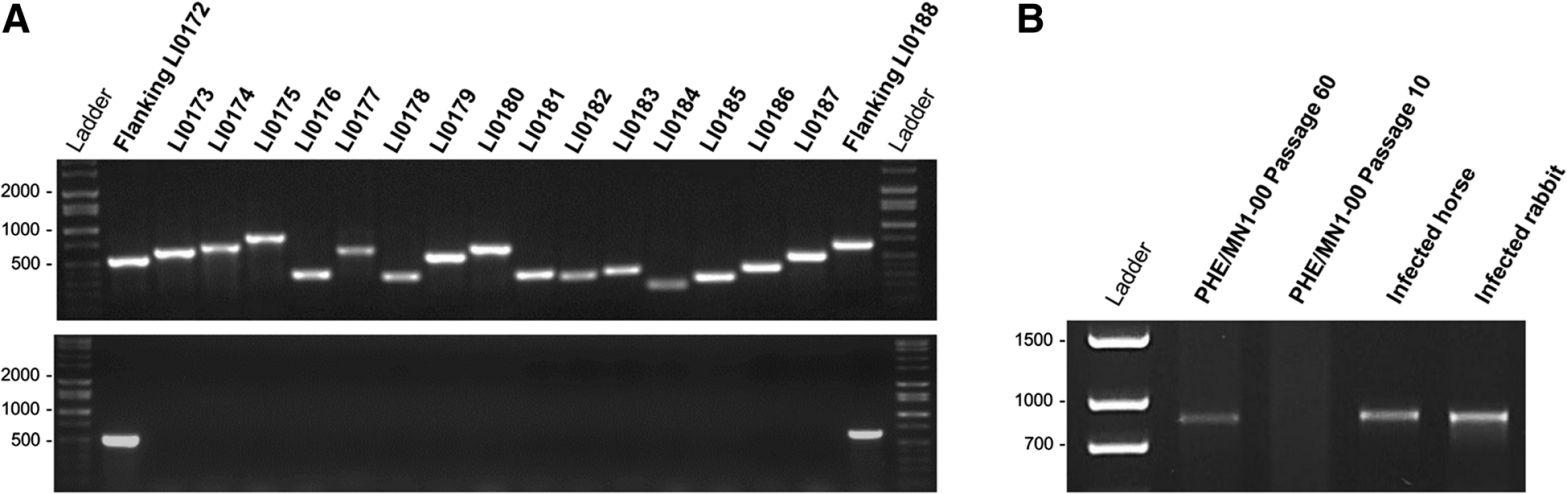
**PCR detection of the prophage DLP12-associated genomic island. (A)** PCR panel for detection of all 15 genes included in the prophage region and the two flanking genes. Porcine *L*. *intracellularis* isolate PHE/MN1-00 at passage 10 showing the presence of all prophage-associated genes (above). Loss of the prophage element at passage 60 showing the presence of flanking genes (below). **(B)** Absence of the entire prophage-associated island confirmed in the porcine isolate PHE/MN1-00 at passage 60 and infected horse and rabbit isolates by obtaining PCR products from the amplification of a primer pair targeting the regions immediately before and after the prophage region (forward: 5′-TCGTGAGAAACTTGTATCAATCCA-3′ and reverse: 5′-TGACAATGTTAGAGCAATGACTTTTTA3-3′).

The presence of all 15 genes included in the prophage-associated genomic island was confirmed by PCR and Sanger-based sequencing (Figure 2A). The loss of this genomic island in the passage 60 was confirmed by the absence of amplified products from all 15 genes and the presence of the amplified product targeting the flanking regions immediately before and after the prophage region (Figure 2B). The prevalence of the prophage-associated island in *L*. *intracellularis* isolates and infected animals was evaluated based on the PCR panel targeting all 15 genes within the genomic region described in Figure 2. In those bacterial isolates or infected animals that harbor this genomic island all 15 genes were found. Its absence was represented by an absence of the entire region. This observation characterizes the chromosomal island as a mobile genetic element.

### Prevalence of the prophage-associated genomic island in *L*. *intracellularis* isolates

The presence of the prophage-associated genomic island was evaluated in a total of 13 *L*. *intracellularis* isolates, ten from pigs, two from horses and one from a hamster (Table 1). Regardless of the origin of the bacterial isolate (North America, South America or Europe), porcine isolates at low passages in vitro (up to 20) show the presence of the prophage-associated island. However, this genetic element was entirely lost in all porcine isolates passed more than 40 times in cell culture (Table 1).

A distinct scenario was observed in the two equine isolates (low and high passage) which do not harbor this genomic island regardless of the number of passages in vitro. Although the pathogenicity of the equine isolate at low passage (E40504) has been demonstrated in previous studies [21,22], the prophage island was not found even in lower passages (four, six and eight) of this isolate (data not shown).

### Prevalence of the prophage-associated genomic island in infected animals

A total of 26 porcine samples that previously tested positive for the presence of *L*. *intracellularis* DNA in the feces were evaluated, derived from seven states of the United States (Iowa, Illinois, Minnesota, Missouri, Nebraska, Oklahoma and North Carolina). Twenty-two samples were derived from pigs affected with the chronic form of PE and four from pigs affected with the acute form of the disease, known as proliferative hemorrhagic enteropathy (PHE). Regardless of the clinical presentation of the disease or the state of origin, all the porcine-derived samples consistently exhibited the prophage-associated island characterized by the presence of all 15 genes within the chromosomal region.

Twenty-one horse samples that were positive for *L*. *intracellularis* by fecal PCR from eight states (Florida, California, Iowa, Kentucky, Minnesota, Tennessee, Texas and Virginia) were evaluated. None of the genomic island genes were present in any equine-derived samples, confirming the observations from the two equine isolates previously described in this study. The absence of the prophage island also was observed in samples obtained from four wild rabbits captured in a horse breeding farm experiencing clinical cases of PE.

## Discussion

Comparing the whole genome sequences of a porcine *L*. *intracellularis* isolate cultivated for 10 and 60 passages in vitro, we identified the loss of a prophage-associated genomic island in the passage 60. This chromosomal deletion comprises a total of 15 genes (18 086 bp) starting in the defective prophage DLP12 (a defective lambdoid prophage at the 12^th^ minute of the chromosome) previously described in *E*. *coli*[23]. The full length of the prophage DLP12 in *E*. *coli* K-12 contains 22 genes included in a region of 21 302 bp [24]. From an evolutionary perspective, this evidence suggests that the element was partially acquired by *L*. *intracellularis* or underwent a partial deletion once integrated into the *L*. *intracellularis* genome. While DLP12 genes in *E*. *coli* encoding a two-component lysis cassette (holing-endolysin) are responsible for inducing lysis of the host bacterial cell, this cassette was not found in the prophage DLP12-associated island of *L*. *intracellularis*. As a result, this defective prophage integrated into *L*. *intracellularis* may have lost a function essential for lytic growth and can no longer liberate infectious particles.

Although the impact of the prophage DLP12 in cell physiology and virulence remains unclear, studies have associated the presence of this integrated prophage with resistance to environmental stress and biofilm formation [24,25]. Rhodius et al. showed activation of DLP12 genes mediated by the transcription regulatory factor σ^E^ during the stress response [26]. A recent study demonstrated the role of the DLP12 genes in maintenance of the bacterial cell wall and in biofilm development by curli-producing *E*. *coli*[25]. To date, no information has been presented regarding the ability of *L*. *intracellularis* to produce biofilm. However, empiric observations of recurrent clinical infections in certain swine production systems may support future investigations regarding biofilm production, based on the potential persistence of *L*. *intracellularis* in swine installations and/or fomites. Previous studies did not identify expression of the 15 genes included in the prophage-associated island by *L*. *intracellularis* in the host cytoplasm in vitro or in vivo [27,28]. Therefore, similar to curli-producing *E*. *coli*, the expression of the DLP12 genes in *L*. *intracellularis* is likely to occur during the stationary phase of the extracellular life cycle.

The authors identified the persistence of the prophage DLP12-associated element for at least 20 passages in vitro in two porcine isolates and its loss after 40 passages in cell monolayer (Table 1). The low excision frequency reported for this prophage in *E*. *coli* K-12 (<1 per 100 000 cells) may explain the gradual loss of this genetic element during *L*. *intracellularis* cultivation in cell monolayer [24]. During the cultivation of *L*. *intracellularis* in vitro the infection is passed weekly [6], which gives the prophage DLP12 a stability of at least 20 weeks. The excision rate of integrated phages is directly related to the adverse conditions encountered by the host bacterial cells followed by the induction of SOS response in the bacteria and conversion of the phage from its lysogenic to lytic form [29]. However, prophage DLP12 in *E*. *coli* lost the ability to excise and form phage particles on SOS response challenge [24]. This defective prophage may now suffer the same fate as its host. The beneficial impact of defective prophages on cell physiology includes enhancing nutrient utilization and increasing the host’s tolerance to general environmental stress and antibiotics. Therefore, the loss of this element does not compromise essential host physiological functions and it becomes dispensable under standard culturing conditions [24]. Additionally, the role of the DLP12 genes in cell wall maintenance and composition suggests an important means for bacteria to improve their fitness. It has been theorized that introduction of novel genes by phages can confer beneficial phenotypes that allow the exploitation of competitive environments [30].

A gene encoding ribosomal protection protein associated with tetracycline resistance was found in this prophage-associated island. Although the wide use of antimicrobials in the swine industry over the years has been speculated to select resistant and/or virulent bacterial strains [31,32], the phenotypic consequence of this tetracycline resistance gene in *L*. *intracellularis* infections is unclear. Wattanaphansak et al. evaluated the antimicrobial activity in vitro of chlortetracycline against ten porcine *L*. *intracellularis* isolates which originated from North America and Europe [33]. Regardless of the geographical origin and the number of passages in culture, which ranged from 7 to 170, the authors found a large variation among these bacterial isolates in regard to their susceptibilities to chlortetracycline [33]. Though resistance to chlortetracycline in low passages could be due to the presence of the tetracycline resistance gene in the prophage island, low passage isolates tested in that study did not consistently demonstrate resistance to chlortetracycline. This study also showed a higher antimicrobial activity of chlortetracycline against intracellular *L*. *intracellularis* compared with extracellular organisms. Associating this information with the lack of expression of this tetracycline resistance gene by *Lawsonia*, especially by intracellular organisms, and the properties of DLP12 prophage to cope with environmental stress (previously discussed), it is possible that this gene may be expressed extracellularly and contribute to a higher antimicrobial tolerance during the extracellular stage. However, this hypothesis needs to be specifically addressed in a future study.

Despite the limited number of *L*. *intracellularis* isolates available worldwide, our results showed the presence of the entire prophage region in ten porcine isolates at low passage *in vitro* indicating the consistent adaptation and specificity of this genomic island to porcine-derived isolates, regardless of the clinical form (acute or chronic) of the disease. The absence of this genomic island in the equine isolate at low passage reveals an important genomic variation related to species-specificity of *L*. *intracellularis* isolates. The availability of only one hamster isolate that has been cultivated over 100 times in vitro restricts a more confident conclusion regarding the prevalence of the prophage in this species. Nevertheless, this is the first study describing a significant genomic variation among *L*. *intracellularis* isolates, since to date *L*. *intracellularis* has been considered a monotypic organism [34].

In addition to the porcine isolates, the presence of the prophage DLP12-associated genomic island was confirmed in fecal samples from PE-affected pigs and its absence confirmed in horses and rabbits infected with *L*. *intracellularis*. Despite the fact that the loss of this genetic element was coincident with the loss of virulent phenotype in one porcine isolate (PHE/MN1-00) [13], the consistent lack of this island in infected horses shows that this prophage-associated island is not essential for virulence in *L*. *intracellularis* infections. However, our results show that this genomic region is indeed associated with host-adapted *L*. *intracellularis* variants.

Supporting the genotypic variation among the species origin of the bacterial isolate described here, a previous study phenotypically demonstrated the host adaptation of *L*. *intracellularis* in pigs and horses [35]. In a cross-species experimental model using pure culture of *L*. *intracellularis* at low passages, pigs and foals infected with their species-specific isolates shed the bacterium in the feces for longer periods of time and exhibited stronger serologic immune responses compared with foals infected with the porcine isolate and pigs infected with the equine isolate. While the porcine isolate in the referred study harbors the prophage, the equine isolate does not have this genetic element. This evidence of host adaptation in pigs and horses seems to extend to experimental infection models using hamsters and rabbits [36]. Sampieri et al. showed that rabbits are more susceptible to equine-derived isolates than to porcine variants and hamsters more susceptible to porcine-derived isolates than to equine variants [36]. These results suggest that *L*. *intracellularis* variants evolved to be adapted to more than one host species. The evolutionary ecology responsible for driving host-adapted variants remains to be determined. Although our study failed to specifically associate the prophage DLP12 genomic region with virulent phenotype, we hypothesize that this genetic element contributes to the adaptation of *L*. *intracellularis* in the porcine host. Based on evidence showing the role of this defective prophage in helping *E*. *coli* cope with adverse environment and form biofilms, this genetic element may drive an ecological specialization in pig-adapted *L*. *intracellularis* variants. In a future perspective, the whole genome sequencing of horse isolates could allow a more comprehensive conclusion regarding the genetic traits related to the host-adapted *L*. *intracellularis* variants. Recently, the analysis of whole genome sequences of *Staphylococcus aureus* ST398 strains derived from livestock and humans showed the presence of the prophage φ3 in human but not animal-derived isolates [37]. The authors suggest that these differences at the genome level are able to drive host-specific adaptation in the ST398 strains. Additionally, the prophage φ3 seems to be lost after jumping from humans to livestock.

In summary, the present study showed a prophage-associated genomic island present specifically in pathogenic porcine *L*. *intracellularis* isolates. The identification of this genetic element may be applied as a molecular marker to determine the species origin of the bacterial isolates and to trace the evolutionary history related to the host species in *L*. *intracellularis* infections. Finally, this first study showing significant genomic variation in *L*. *intracellularis* isolates associated with the previously reported host-adapted phenotype may support the characterization of novel *L*. *intracellularis* subspecies or species-specific genotypes.

## Competing interests

The authors declare they have no competing interests.

## Authors’ contributions

Conceived and designed the experiments: FAV, MRK, CJG. Performed the experiments and analyzed the data: FAV, MRK, CJG. Wrote the paper: FAV, MRK. Coordinated and helped to draft the manuscript: CJG. All authors read and approved the final manuscript.
